# Editors’ Book Table

**Published:** 1875-06

**Authors:** 


### Editors' Book Table.

[Note.—All works reviewed in the pages of the Chicago Medical Journal may be found in the extensive stock of W. B. Keen, Cooke & Co., whose catalogue of Medical Books will be sent to any address upon request.]

Cyclopædia of Practical Medicine. Edited by *Dr. H. Von Ziemssen*, Professor of Clinical Medicine in Munich, Bavaria. Vol. II. Acute Infectious Diseases. By Prof. Thomas, of Leipzig, Dr. Curschman, of Berlin, Dr. Zuelzer, of Berlin, Prof. Hertz, of Amsterdam, and Prof. Ziemssen, of Munich. Translated by James C. White, M.D., and Edward Wigglesworth, Jr., M.D., of Boston, Edward W. Schauffler, M.D., of Kansas City, and A. Brayton Ball, M.D., J. Haven Emerson, M.D., George H. Fox, M.D., Edward Frankel, M.D., and John C. Jay, Jr., M.D., of New York. Albert H. Buck, M.D., New York, Editor of American Edition. New York: William Wood & Co. 1875.

The flattering commendation so generally bestowed by the American medical press, and the hearty welcome accorded by the profession to the first volume of Ziemssen's Cyclopædia, warranted the expectation that the publishers would be encouraged to issue the succeeding —the second—volume at an early day. It is gratifying to perceive that these anticipations have been so speedily realized, and the second installment of "Acute Infectious Diseases" is at hand.

The discussion of varicella, measles, *rubeola* and scarlet fever, is from the pen of Prof. Thomas, of Leipzig, who seems to have acquired, at an early period of life, an extensive reputation on the other, although hitherto unknown upon this, side of the ocean. The treatise upon measles is voluminous, and the literature of the subject, as far as it is comprised in the works of German authors, exhausted. A few Dutch and French authorities appear to have been referred to, but only a very few English writers, and these not of the more recent, seem to have been consulted, while, as in the first volume, American authorities are entirely ignored, with the exception of Drake, who is quoted once, and Salisbury. Regarding the etiology of measles, it is satisfactory to perceive that the author has not been carried away by the spore-theory of contagion, which appears to have captivated so many of his countrymen. Regarding its diagnosis in colored races, he asserts, upon the authority of Praver, that the eruption upon negroes "is distinguished by little vesicles." These were in all probability examples of intercurrent herpes, as the reviewer has had the opportunity to examine a large number—approximating one hundred—cases of this disease in negroes, in whom the eruption was invariably black; that is, much blacker than the prevailing tint of the negro's skin. In reference to the "measles-like eruption of dengue," there can be no liability to confusion to one at all familiar with the two diseases, the eruption of dengue resembling in no respects that of measles, as we have had the opportunity to verify on many occasions. No reference is made to the anæsthesia, especially paraplegic and gustatory, so characteristic of dengue. Concerning the treatment of measles, it will strike an American practitioner as a novelty that for the "suppression of immoderate fever in the prodromal, and especially in the eruptive stage," "at present, cool baths, packings and extensive cold compresses are the usual means employed."

Is this the beginning of a new era of hydropathy? Or is it a new phase of the temperance movement?

The author rejects the term rubeola as a synonym of measles, claiming for it a specific identity with Rotheln, German measles, roseola or rougeole, and asserts, and we believe correctly, that this is the true nature of many of the so-called "second attacks" of measles.

The treatise upon scarlatina is from the pen of the same author, and consists of a very brief historical sketch, followed by an interesting paper upon the etiology of the disease, in which, in referring to the experiments of Hallier and Riess, the author concludes that "these experiments therefore render it highly probable that the contagious principle, having entered the blood, is disseminated by it throughout the body; and further, that it stands in some very intimate relation with these finest spores or micrococcus." Concerning the influence of social position, he asserts "that the mortality increases with poverty and decreases with affluence, though its dependence on the latter is not so apparent as in the case of measles;"—conclusions which experience in this country will scarcely justify. "Conditions of soil," he thinks, may also occasionally exert an influence, as likewise does "a residence in certain localities." The influence of age as a predisposing cause of the disease, is established by copious statistical tables. He thinks, too, that "those who have been operated upon, and the wounded," possess "a greater susceptibility to the disease." Influence of race he considers as undecided. He admits the possibility of second attacks of the disease, "besides a few reports of a third and fourth infection in the same individual."

The chapter on pathology is full. The affections of the lymphatic glands and the joints, and the diseases of the kidney, together with the characteristics of the urine, receive their due share of attention.

The irregular forms of scarlatina, and its complications, are discussed at large, and are followed by a condensed epitome of its sequelæ.

As regards the treatment of scarlet fever, we most heartily endorse all that the author has to say upon the subject of prophylaxis, which may be summarized thus —isolation, ventilation, disinfection, and thorough cleanliness. The "symptomatic," he claims, is the only rational and advisable treatment for scarlet fever; and while his commendation of hydro-therapeusis will receive the cordial endorsement of many, his frequent citations of Priessnitz suggest to the American reader the query, "Can any good come out of Nazareth?"

Dr. Curschman's treatise on small-pox consists of a summary of his own conclusions, deduced from personal observation of the disease, in the recent epidemic in Mayence, in 1870–71, and his investigations into the literature of this malady, from its earliest history down to the present time. Discrediting earlier accounts, the author considers as the earliest advent of the disease into Europe, the epidemic of 581, described by Gregory, of Tours, and the "first scientific description of it, that of Constantine Africanus," of Salerno. Noting briefly its ravages in Europe, he refers to its spread thence to America, and its frightful ravages in Mexico, in 1527, and considers that the virulence of the disease was unabated by any remedial measures until the introduction of vaccinia by Jenner. The author regards, and justly, perhaps, the inoculation of the small-pox poison as of questionable benefit, inasmuch as, while it undoubtedly modified the intensity of the morbid phenomena in the individual subject, it at the same time multiplied morbid foci, from which it was capable of infinite radiation and reproduction.

The author offers no conjectures upon the subject of the etiology of small-pox, but furnishes some statistics of the epidemic at Mayence, showing the relative susceptibility at different ages, together with a number of examples of the occurrence of the disease in extremely early periods of intra-uterine life, as early even as in the fourth and fifth months.

Regarding what might be termed its collateral morbid relations, he says: "It can be asserted with certainty, *that for an individual suffering from scarlet fever, measles or typhoïd fever, there is, during the entire duration of the affection, a very slight liability to an attack of variola only;*" which, after all, seems to be asserting very little "with certainty," and, as far as regards measles, at least, the assertion will not be received with absolute certainty by some observers.

As to the liability o second infections of small-pox, the author is skeptical, although citing the example of Louis XIV, who died from a second attack of the disease, just fifty years after the occurrence of the first. He claims the purely specific nature of the virus, its residence in the pustule—although admitting the possibility of infection by inoculation of blood—its transmissibility by means of "perspiration" and "gaseous particles," and "the atmosphere about the patient," which, "imparting the contagion" to surrounding objects, "clings to them for a variable length of time." He asserts that "small-pox virus impregnating gaseous media, and excluded from the air, is possessed of very great permanence;" that "the contagion" is "readily destroyed" by exposure "to atmospheric air," but that high temperatures, the vapors of chlorine, iodine, bromine, sulphur and alcohol, act deleteriously upon the contagion only after very intense and long-continued influence.

The terms infection and contagion are used very loosely; sometimes apparently as synonyms, again distinctively, and sometimes objectively, to indicate a matines morbi, and again subjectively, designating the act of transmission of the same.

It is impossible to acquire any definite impressions upon this subject of epidemiology until writers shall have determined the correct etymology of the technical language in which their propositions are couched. A translator would seem to be warranted in so far violating literal fidelity as to correct such ambiguities.

In the short paragraph relating especially to pathology, the author's definition of the relations of variola and varioloid is by no means as clear as is desirable. "No one any longer considers varioloid as an independent disease, as was formerly zealously maintained. Every one knows that we see nothing further in this affection than one of the many forms in which small-pox is manifested, according to circumstances." In view of the universal belief in the essential relations of varioloid to vaccinia, it would have been better had this proposition been more restricted in its comprehension.

The period of incubation of small-pox, the author fixes "most frequently at from ten to thirteen days, less frequently fourteen days, or eight to ten days, and in one case only five days;" the "initial stage" at "three days," "as a rule;" the temperature "on the first day" "often" 103° to 104°, "on the evening of the second or third day, frequently" 105° to 105½°, and in some cases "even above 107°." The observations of temperature were made "in the axilla," which, by the way, will be found very frequently to furnish a higher range of temperature than the mouth.

The symptomatology of the disease is given very fully and satisfactorily; but we must enter a demurrer to the proposition of the author, italicized upon page 373: "It is generally conceded that varioloid is nothing more than a form of small-pox, with a milder course and a shorter duration." Whatever may be the fact in Germany, no such general concession has been made in America, and the assertion will, we think, strike most of our readers with surprise.

From this he proceeds, quoting Hebra, to describe several of these various forms of variola or varioloid, and then briefly to note the complications of the disease, after which he gives a well written sketch of its morbid anatomy.

The difficulty of early diagnosis, and the liability to confound the disease with syphilitic eruptions and acne pustulosa, are fully recognized, although no reference is made to a possible source of doubt which might occur in the eruption sometimes observed to follow the use of balsam copaiba, and which has been actually diagnosed as that of variola. "One pathognomonic symptom" is indicated, although "present only in the smaller number of patients. It consists in the hæmorrhagic initial exanthema, situated principally in the triangle of the thigh."

The mortality from small-pox is said to have diminished, since the last century, from 7 to 12 to 0.7 to 1 per cent. of the total.

As a prophylactic, the author's confidence in vaccination is thorough; but he has no confidence in its protective capacity when performed during the stage of incubation. Abundant evidence exists on this side of the ocean to demonstrate that the protective power of vaccination is efficient even during this period of incubation. The possibility of securing this protecting influence is suggested by the difference in the length of the period of incubation of the two diseases, that of vaccinia being two, while that of variola is generally from "ten to thirteen days." This suggestion has been practically and successfully acted upon so often as to leave no room for doubt upon the subject. The author's ideas on the subject of treatment are rational and judicious. He does not try to "cure the disease," but to keep the patient alive, and as comfortable as possible under the circumstances, and has no faith in efforts at abortion either of the disease or the eruption.

The discovery of vaccination is, justly, assigned to Jenner, and his merit is by no means diminished by the pre-existence of the knowledge that persons who had contracted the vaccine disease enjoyed an immunity against small-pox. This fact constituted the essential premise of Jenner's induction, without which it could not have been made.

The origin of vaccinia is attributed to a similar disease occurring in horses, and the existence of manifestations of the same character in other animals susceptible of intercommunication, is asserted.

Dr. Curschman dissents from the belief in the deterioration of humanized vaccine, and perceives no advantages in the use of non-humanized virus. He is very justly skeptical concerning the transmission of constitutional diseases, more especially syphilis, by vaccination, and with equal justice attributes the very rare instances in which such results have occurred, to carelessness in the selection of the subject and of the lymph, and in the performance of the operation. He is a warm advocate of compulsory vaccination by State interference—a proposal which presents to Americans two phases so strikingly contrasted as to necessitate great deliberation in its adoption or rejection. The subject of re-vaccination is entirely ignored, except incidentally.

Taken altogether, Dr. Curschman's article is an excellent compendium of the state of knowledge upon the subject of which it treats, and will constitute a very useful work for the general practitioner. H.

ESSAYS ON CONSERVATIVE MEDICINE AND KINDRED TOPICS. By *Austin Flint, M.D.*, Professor of the Principles and Practice of Medicine and of Clinical Medicine, in Bellevue Hospital Medical College, New York. Philadelphia: Henry C. Lea. 1874.

Prof. Flint's name has been so thoroughly identified with conservatism, that it appears with peculiar propriety upon the title page of a volume devoted to its exposition. And the presentation of the thoughts and opinions of so accomplished a physician and so ripe a scholar, in this form, is a contribution to the literature of the profession which may exert upon it ultimately an influence as beneficial as have some even of his more practical works. Every young man should read these "Essays," for he cannot fail to derive both pleasure and profit; and men of longer professional life and larger experience will likewise find perhaps some of their best thoughts formulated into language their best judgment confirmed by the conclusions of the author. The little book will become one of the classics of professional literature. H.

On Functional Derangements of the Liver, Being the Croonian Lectures, Delivered at the Royal College of Physicians, in March, 1874. By *Charles Murchison, M.D., LL.D., F.R.S.*, Fellow of the Royal College of Physicians, Physician and Lecturer on the Principles and Practice of Medicine, St. Thomas Hospital; Vice-President and Consulting Physician, London Fever Hospital; formerly Physician and Lecturer on Medicine, Middlesex Hospital; and on the Medical Staff of H. M.'s Bengal Army. New York: William Wood & Co. 1875.

The notice of Dr. Croon, the founder of lectures of which this little book comprises one course, constitutes a graceful introduction to a series of admirable lectures, for the preparation of which the author's experience seems to have especially qualified him. The course consists of three lectures, of which the first is occupied with the physiology of the organ, which are formulated under three heads.

1st. The formation of glycogen, which contributes to the formation of animal heat and to the nutrition of the blood and tissues; and the development of white blood-corpuscles.

2nd. The destructive metamorphosis of albuminoid matter, and the formation of urea and other nitrogenous products which are subsequently eliminated by the kidneys, these chemical changes also contributing to the development of animal heat.

3rd. The secretion of bile, the greater part of which is reabsorbed, assisting in the assimilation of fat and peptones, and probably in those chemical changes which go on in the liver and portal circulation; while part is excrementitious, and in passing along the bowel stimulates peristalsis and arrests decomposition.

It will be perceived that the author very justly rejects Liebig's theory of the production of animal heat exclusively to the destructive metamorphosis of carbonaceous ingesta, in the adoption of which that great chemist seems to have so strangely overlooked one of the most obvious results of the liberation of force by the disturbance of chemical affinities.

In the second portion of this chapter, devoted to "functional derangements of the liver," the relations between the hepatic and renal functions, as illustrated in the development of diabetes, are clearly defined. In estimating the importance of cholesterine as a pathological factor, the author differs from Dr. Austin Flint, Jr., arguing that "in cases of permanent closure of the bile-duct, cholesterine is not discharged from the liver into the bowel; it does not accumulate in the biliary passages, nor, if it be present in the blood, does it necessarily give rise to cerebral symptoms." Hence, "if the non-excretion of all the elements of the bile does not give rise to cerebral symptoms, it is difficult to understand how these symptoms can result from the retention of cholesterine alone." On page 120, the author endeavors to account for the "cerebral symptoms" symptomatic of certain morbid conditions of the liver, by the presence of "leucin and tyrosin," "deleterious products of disintegrating albumen," thereby apparently placing himself in the same dilemma which he assumes to be occupied by Flint. The author proposes a new system of classification of "Functional Derangements of the Liver," *i.e.*: 1, Abnormal Nutrition; 2, Abnormal Elimination; 3, Abnormal Disintegration; 4, Derangements of the Organs of Digestion; 5, Derangements of the Nervous System; 6, Derangements of the Organs of Circulation; 7, Derangements of the Organs of Respiration; 8, Derangements of the Urinary Organs; 9, Abnormal Conditions of the Skin. Which appears to be unnecessarily extensive, the first three classes being evidently susceptible of reduction to one, from their essential correlation.

In considering the therapeutic action of mercury, the author avoids assuming a partisan attitude in reference to the cholagogue or acholagogue effect of the drug, assuming a position which, contradicting the experiments of neither side, may harmonize with both—*i.e.*, that mercury acts by unloading the portal blood of the waste matters contained therein, and also by facilitating the disintegration of albumen.

The book deserves even a more extended notice than has been attempted here, did time and space serve for the purpose. It certainly deserves to be read by all, and if read carefully, cannot fail to be highly appreciated. Not the least of its merits is the style of its publication, which is excellent, the clear type and fine paper being very attractive to the eye of a lover of good books. H.

TRANSACTIONS OF THE TWENTY-FOURTH ANNIVERSARY MEETING OF THE ILLINOIS STATE MEDICAL SOCIETY, Held in the City of Chicago, May 19th, 20th, 21st, 1874.

The transactions comprise various addresses, appropriate to such occasions, reports upon surgery, on galvano-therapeutics, on muriatic acid in the treatment of continued fever, on malarial diseases and their treatment, on continued immersion in compound fractures, in lacerated and in pointed wounds, contributions to the report on practical medicine, report on stricture of the urethra, on necrology, on otology, on orbital tumors, and on idiocy. The report upon surgery commends highly bloodless surgery, as accomplished by the process of Esmarch, which it regards as the inauguration of a new era in practical surgery, equal in importance to those which dated from the first ligation of an artery by Ambroise Pare, and the first joint-resection. The report is equally commendatory of pneumatic aspiration, and gives the preference for the performance of this operation to the aspirator of Mathieu, Paris. The report is especially enthusiastic on the subject of the Bavarian or bi-valve splint of plaster of Paris, for the formation of which minute directions are given. Concerning anæsthetics, after a review of the Boston case of Mrs. Crie, and quotations from the experts examined on that occasion, the reporter admits the "growing conviction that the use of chloroform ought to be abandoned for the purposes of anæsthesia," and that he is himself "compelled somewhat sorrowfully to share in this conviction." He attributes the impunity with which chloroform was administered by army surgeons, during the war, to the fact of the customary previous administration of whisky. It seems strange that observers of the effects of anæsthetics should appear to have directed their attention exclusively to the condition of the heart, overlooking therein the modifications of innervation, proximately induced by the influence of these agents, and of which the changes observed in the condition of the heart are but secondary and remote consequences. The first effects of chloroform are undoubtedly manifested upon the nervous system, and assume the form of increased arterial tension, the direct result of vaso-motor irritation. An extension of this irritation, and resulting arterial tension, is soon apparent in the cerebral circulatory system, as indicated by the dilatation of the pupil and the extreme pallor of the now almost pulseless patient, whose heart still beating awaits the extension of this irritation to the pneumogastric nerve, to still its throbs in death.

The successful application of the postural treatment to the restoration of patients syncopated by chloroform, demonstrates clearly the truth of the above proposition, the rationale of the treatment being simply the introduction by the operator of another mode of force, gravity, (Mayer's "falling force") to the restoration of the equilibrium between arterial and cardiac action, disturbed by the influence of the chloroform acting as a vaso-motor stimulant. The heart, overwhelmed by the combined influence of the vis a fronte, arterial spasm, and of the vagus nerve, can no longer propel a sufficient amount of blood to the brain to maintain therein the degree of stimulation necessary to continue the circulatory process, and bring back to itself blood enough to maintain its own accustomed stimulus. From such a condition, escape is impossible by means of any auto-genetic agency. If the patient is now inverted, and maintained in that position, a new factor is introduced from without, gravity, under the influence of which the arterial tension is overcome by hæmostatic pressure, the blood descends in increased quantity into the vessels of the inverted brain, an increased supply of nerve force is developed, the pupils contract, the inhibitory influence of the vagus is overcome, more blood is admitted to the heart, this organ is awakened to renewed life, and with its restored activity the pulse beats anew, color and warmth return, and the normal equilibrium of function is restored.

The reports generally deserve extended notice, did time and space permit—none of them, perhaps, more so than that on Ophthalmology, by Prof. E. L. Holmes, and that upon Idiocy, by C. T. Wilbur. The reading of the proof has not been as carefully performed as it might have been, as is rendered apparent by numerous orthographical errors, which could scarcely be attributed to the authors of the papers. Taken altogether, the volume is exceedingly creditable to the members of the State Medical Society. H.

The Complete Hand-Book of Obstetric Surgery. By *Charles Clay, M.D.*, Late Senior Surgeon and Lecturer on Midwifery, St. Mary's Hospital, Manchester, England. Philadelphia: Lindsay & Blakiston.

The design of the author in this little book is implied in the title. While we have read the book with some interest, frankly, we cannot see the necessity of hand-books in any department of medicine; and least of all do they seem called for in obstetric surgery. Is it intended as the first book for a student? What student would be satisfied with the incomplete description of obstetric operations and diseases which a small hand-book must necessarily contain! If unsuitable as a first book, it is not necessary as a review, which can be made in a completer treatise. Is it intended for the busy practitioner? What surgeon would perform ovariotomy with no other book than this to recall to his mind the many points in the operation! If it is convenient for the practitioner to consult this book in an emergency, it will be equally convenient for him to consult a completer monograph. Still, any one who has plenty of time and means to devote to books of all kinds, may also read this with some profit.

The arrangement of the subjects, which the book contains, in alphabetical order, may present some advantages, yet it seems like going from extreme to extreme to speak of the treatment of piles and hare-lip upon the same page.

The author says of chloroform as an anæsthetic agent, "we have no other that offers the same immunity from suffering." We believe this, the extension of an error; for every surgeon and obstetrician in America knows that sulphuric ether gives an immunity from pain quite as complete, while its use is infinitely less hazardous.

Further, "it is a singular and significant fact, that no death has been recorded from its use in midwifery;" this he attributes to, *first*, "That the patient is not (or at least ought not to be) completely thrown over into a state of perfect anæsthesia (or snoring), but kept just within the limit; not quite unconscious, yet not sensible to pain." This leads to the inference that an agent acknowledged to be extremely dangerous in surgery may be used with safety in obstetrics, attention being drawn only to the *degree* of anæsthesia. This is wrong. It may be that no death has been *recorded* from the obstetric use of chloroform, but at least two instances have been narrated to us of women who have died in labor, from the effects of chloroform, in the hands of gentlemen experienced in its use. It is a fact, unfortunately, that death from chloroform occurs oftenest before the state of insensibility is reached, and when the victim is in just that state which the author urges is safe. The conclusion is, that chloroform is endowed with a property, peculiar to itself, which will surely destroy life under some circumstances which we are not able to recognize; further, in some instances, the inhalation of a few drops *only* is sufficient to kill. S.

### PAMPHLETS RECEIVED.

THIRTEENTH BIENNIAL REPORT of the Illinois Institution for the Education of the Blind, for 1873, 1874, 1875.

ANNUAL REPORT of the Supervising Surgeon of the Marine Hospital Service of the United States for the Fiscal Year 1874. *Jno. M. Woodworth, M.D.* Washington. 1874.

### JOURNALS RECEIVED.

The American Medical Weekly—Vol. ii, Nos. 17, 18.
The Archives of Dermatology—Vol. —, No. 3.
The American Practitioner—Vol. xl, No. 65.
The Canada Medical and Surgical Journal—Vol. iii, No. 10.
The Cincinnati Lancet and Observer—Vol. xviii, No. 5.
The Chicago Journal of Nervous and Mental Disease—Vol. ii, No. 2.
The Clinic—Vol. viii, Nos. 17, 18, 19.
The Detroit Review of Medicine and Pharmacy—Vol. x, No. 5.
The Dental Cosmos—Vol. xvii, No. 5.
The Eclectic Medical Journal—Vol. xxxv, No. 5.
The Indiana Journal of Medicine—Vol. vi, No. 1.
The Journal of Materia Medica—Vol. xiv, No. 5.
Kin Le I Lelzin. Yokohama, Japan.
The Laboratory, Boston—Vol. i, No. 10.
The London Lancet, April, 1875.
The Medical Times (Philad.)—Vol. v, Nos. 183, 184.
The Medical and Surgical Reporter—Vol. xxxii, Nos. 17, 18, 19.
The Medical Record—Vol. x, No. 17.
The Medical Press and Circular, London—No. 1838.
The Monthly Abstract of Medical Science—Vol. ii, No. 5.
The Medical News and Library—No. 389.
The Nashville Journal of Medicine and Surgery—Vol. xxxvi, No. 5.
The New York Medical Journal—Vol. xxi, No. 5.
The Ohio Medical and Surgical Reporter—Vol. ix, No. 3.
The Pharmacal Gazette—Vol. iii, Nos. 65, 66, 68.
The Practitioner, London—No. lxxxii.

The Peninsular Journal of Medicine—Vol. ii, Nos. 4, 5.
The Progres Medical, Paris, 3c Annee—Nos. 11, 12, 13, 14, 15.
The Pharmacist, Chicago—Vol. viii, No. 5.
The Psychological and Medico-Legal Journal—Vol. 2, No. 4.
The Southern Medical Record—Vol. v, Nos. 2, 3.
The St. Louis Clinical Record—Vol. ii, No. 2.
The St. Louis Medical and Surgical Journal—Vol. xii, No. 5.
The Virginia Medical Monthly—Vol. ii, No. 2.

---

Compendium der Neuesten Medizinishen Wissenschaften. By *Dr. B. Kraus.* Pages, 832. Vienna: Sporitz Perles.

Having been requested, by the author, to write a short criticism on the above work, I comply hereby with his wish rather reluctantly. The author is chief editor of the "Allgemeine Wiener Medizinishe Zeitung," a medical weekly which stands deservedly high, for the really valuable articles it contains, mostly written by men of eminence, as for instance, Billroth, Pitha, etc. The Compendium comprises the auxiliary sciences to the study of medical diagnosis, viz.: Thermometry, Sphygmography, Percussion and Auscultation, Microscopy, Uroscopy, and the special branches, as Laryngoscopy, Anomalies of Speech, Electro-Therapeutics, Hygiene, and Toxicology. The articles on the different topics are written in a concise style, though sufficiently exhaustive for practical purposes. They are mostly compiled by the author himself. At the end of each article, he gives a short bibliography used by him.

As I consider no medical work on any subject complete which does not refer to standard American authorities, the shortcomings of the Compendium are apparent. Austin Flint, Da Costa, Meigs, Pepper, Jacobi, and a good many others who have contributed to some of the above medical branches, are not mentioned. If a future edition should become necessary, the author will do well to correct this deficiency.

Anomalies of Speech, written by the well-known Dr. Coen, an authority in that especial branch of medical science, is treated in an excellent manner, as might be expected.

The article on Electro-Therapeutics comes from the pen of Staff-Surgeon Sewandowsky, treats the subject fairly, and quotes our countrymen, Beard and Rockwell, quite often. Toxicology and Hygiene emanates from the pen on the author's brother, Dr. Leopold Kraus; and as no one can expect in sixty pages but a very short synopsis on those topics, the author has done justice to the subjects under consideration, in a very creditable manner.

The work, taken as a whole, reflects credit on its author, and deserves to be read by every physician who wants information on those medical subjects, and I have no doubt that a translation, performed by competent men, would meet with a favorable reception in this country.

CARL PROEGLER, M.D.

Addison, Ill., April 20, 1875.

